# Dingkun pill alleviates metabolic abnormalities in polycystic ovary syndrome through brown adipose tissue activation

**DOI:** 10.1186/s13048-023-01215-0

**Published:** 2023-08-26

**Authors:** Mengqing Gu, Han Cai, Weinan Deng, Yedong Tang, Shuailin Du, Peiran Wang, Wenbo Deng, Haibin Wang, Aijun Sun, Shuangbo Kong

**Affiliations:** 1 Fujian Provincial Key Laboratory of Reproductive Health Research, Department of Obstetrics and Gynecology, School of Medicine, The First Affiliated Hospital of Xiamen University, Xiamen University, 361102 Xiamen, Fujian China; 2 Department of Obstetrics and Gynecology, Key Laboratory for Major Obstetric Diseases of Guangdong Province, The Third Affiliated Hospital of Guangzhou Medical University, Guangzhou, China; 3 Department of Obstetrics and Gynecology, Peking Union Medical College Hospital, Chinese Academy of Medical Sciences and Peking Union Medical College, 100730 Beijing, China

**Keywords:** Dingkun Pill (DK), Polycystic ovary syndrome, Brown adipose, Lipid metabolism

## Abstract

**Background:**

Traditional Chinese medicine has been used for a long time to treat a variety of gynecological diseases. Among various traditional Chinese medicine, Dingkun Pill (DK) has been used for the treatment of female gynecological diseases. However, DK therapeutic effect on PCOS and the target tissue for its potential effect need to be explored. This study aims to explore the therapeutic effect of DK for PCOS in mice from three aspects: metabolism, endocrine and fertility, and determine whether the brown adipose tissue is the target organ to alleviate the PCOS phenotype.

**Methods:**

PCOS mouse model was constructed by subcutaneous injection of DHEA. The estrous cycle, ovulation, and pregnancy outcome was examined in mice. The level of hormone including the LH, FSH, estrogen and testosterone in the serum were measured by ELISA. Both the glucose sensitivity and insulin sensitivity were determined in mice with different treatment. The histomorphology and lipid contents in the brown adipose tissue were analyzed. RNA-Seq was conducted for the brown adipose tissue and different expression of critical metabolism marker genes was confirmed by real-time PCR.

**Results:**

The data showed that the fertility in PCOS mice with DK treatment was significantly increased, and the metabolic disorder was partially restored. Both the whiten of brown adipose tissue and reduced UCP1 expression induced by DHEA was rescued by the DK. The RNA-Seq data further demonstrated both the DHEA induced downregulation of lipolysis genes and oxidative phosphorylation genes were at least partially rescued by DK in the brown adipose tissue.

**Conclusions:**

DK has therapeutic effect on PCOS in DHEA treated mice and the brown adipose tissue is at least one critical target organ to alleviate the PCOS. This is achieved by not only regulating the lipid mobilization of brown adipose, but also restoring its thermogenic function.

**Supplementary Information:**

The online version contains supplementary material available at 10.1186/s13048-023-01215-0.

## Introduction

Polycystic ovary syndrome (PCOS) is a common, complex endocrine disorder occurring in women of reproductive age, with a prevalence of up to 15% [1]. It’s usually associated with insulin resistance, hyperandrogenemia, cystic follicles and anovulation or oligoovulation, that being one of the leading causes of anovulatory infertility in women [2, 3]. In addition, patients with PCOS usually display clinical features of irregular menstruation, acne, hirsutism and obesity [4, 5]. However, the pathogenesis of PCOS is influenced by a variety of factors, both environmental and genetic, which makes the causes of PCOS complex and diversified [6]. Thus, this has led to a lack of deep understanding about its pathogenesis, which limits the development of effective therapies for PCOS.

Insulin resistance and hyperinsulinemia are two prominent features of metabolic abnormalities in PCOS. Studies have shown that patients with PCOS are more susceptible to diabetes, cardiovascular disease such as hypertension, hyperlipidemia and infertility due to their hyperandrogenic and insulin resistance [7, 8]. Adipose tissue is mainly divided into white adipose tissue and brown adipose tissue (BAT) [9]. The function of white adipose tissue is to store excess lipids in the form of triglyceride, while brown adipocytes contain a large number of mitochondria, which can be used to generate heat through the specific expressed Uncoupling protein 1 (UCP1) to maintain body temperature. When brown adipose tissue is active, plenty of lipids and glucose will be consumed in this organ [10, 11]. The activation of brown adipose tissue can reduce metabolism-related diseases including obesity [12]. As an important endocrine and metabolic organ, brown adipose tissue is one of the center regulators for the maintenance of systemic metabolic homeostasis. The regulatory molecules (the so-called brown adipokines or batokines) that are released by BAT influence systemic metabolism and convey the beneficial metabolic effects of BAT activation [13]. There has been study reported that brown adipose tissue transplantation can effectively improve metabolic abnormalities and infertility in PCOS rat [14]. Thus, brown adipose tissue could serve as a therapeutic target for the treatment of PCOS.

Chinese herbal medicine has long been used to treat a variety of gynecological disorders in women, such as pelvic inflammatory disease, endometriosis, and uterine fibroids [15, 16]. PCOS has been the subject of numerous studies on the treatment of reproductive diseases with Chinese herbal medicine in recent years. Guizhi Fuling Wan is an herbal formula that has a therapeutic effect on PCOS by reshaping intestinal homeostasis and improving insulin resistance [17]. Additionally, LWDH and Heqi San have a positive effect on the treatment of PCOS dependent on the PI3K/Akt signaling pathway to alleviate insulin insensitivity in muscle tissue and the pancreatic beta cell, respectively [18, 19]. Dingkun pill (DK) is a traditional Chinese medicine that has been used for hundreds of years in the remedy of female gynecological disorders. DK is mainly composed of 30 Chinese medicinal herbs, including *Panax trifolius* L. (Panax ginseng C. A. Mey), *Crocus sativus* L. (Crocus cartwrightianus Herb), *Panax notoginseng (Burkill)* F.H.Chen (Pseudo-ginseng),

*Paeonia lactiflora* Pall. (Paeoniae Radix Alba), *Rehmannia glutinosa* (Gaertn.) DC. (Radix Rehmanniae Praeparata), *Angelica sinensis* (Oliv.) Diels (Angelicae Sinensis Radix), *Atractylodes macrocephala* Koidz. (Rhizoma Atractylodis Macrocephalae), *Lycium barbarum* L. (Barbary wolfberry fruit), *Scutellaria baicalensis* Georgi (Radix Scutellariae), *Cyperus rotundus* L. (Rhizoma cyperi), *Leonurus japonicus* Houtt. (Fructus leonuri), *Conioselinum anthriscoides ‘Chuanxiong’* (Rhizoma corydalis), *Spatholobus suberectus* Dunn (Suberect Spatholobus Stem), *Carthamus tinctorius* L. (Flos Carthami), *Leonurus japonicus* Houtt. (Herba leonuri), *Bupleurum chinense* DC. (Radix bupleuri), *Lindera aggregata* (Sims) Kosterm. (Radix linderae), *Eucommia ulmoides* Oliv. (Cortex Eucommiae), *Zingiber officinale* Roscoe (Zingiberis Rhizoma), *Wurfbainia villosa* (Lour.) Škorničk. & A.D.Poulsen (Fructus Amomi), *Cyathula officinali*s K.C.Kuan (Radix cyathulae), *Neolitsea cassia* (L.) Kosterm. (Cortex cinnamomi), *Glycyrrhiza uralensis* Fisch. ex DC. (Radix Glycyrrhizae Preparata) (http://mpns.kew.org). These herbs have also been fully recorded in the Pharmacopoeia of the People’s Republic of China (2020 Edition). It has significant effects in treating endometriosis, menstrual irregularities and improving the endometrial receptivity [20]. However, its therapeutic effects on PCOS and specific therapeutic mechanisms need to be further explored.

Here, we investigated the therapeutic effects of DK for DHEA-induced PCOS mice. It was uncovered that DK could significantly improve impaired fertility and metabolic abnormalities of PCOS mice. We found that DK activated brown adipose tissue in PCOS mice and regulated the global metabolic homeostasis to alleviate the reproductive disorders in PCOS mice. These evidences provided theoretical and practical supports for the clinical treatment of PCOS with DK.

## Materials and methods

### Animal and PCOS model construction

The 3-week-old C57BL female mouse was purchased from the Laboratory Animal Center of Xiamen University. Five animals per cage were placed in the animal care facilities of Xiamen University, with a 12-hour light and dark cycle under constant environmental conditions. The PCOS mouse model was constructed as previously reported by injecting dehydroepiandrosterone (DHEA) (Sigma, MKCJ5452) subcutaneously for 21 days after weighing at the same time every day, while injecting the corresponding dose of sesame oil with the same number of mice as a control [21]. The Chinese medicine DK in this study was provided by Shanxi Guangyuyuan Chinese Medicine Co., Ltd. (Lot No. 3282301003), and the chemical constituents in vitro and prototypes in vivo of Dingkun Dan has been previously reported [22]. The dose was determined based on the recommended daily intake of humans (2 tablets/60 kg/day) to be 0.0535 tablets/day. All experimental procedures were approved by the Animal Welfare Committee of Research Organization (XMULAC20220184), Xiamen University.

### Estrous cycle detection

The estrous cycle is measured by vaginal smears every day. 15 µl 0.9% normal saline was injected into the vaginal orifice of mice and aspirated back and forth twice, then the mucus was evenly spread onto the glass slides to dry naturally. Wright stain solution (Solarbio, G1040) was added to the glass slides for 3 min, after that it was stained with wright stain solution mixed with an equal volume of ddH_2_O for 5 min. Morphological features of the vaginal epithelium were observed under a microscope to determine the specific stage of the estrous cycle. Nucleated epithelia predominate proestrus, accompanied by a small number of white blood cells. Estrus is basic for seedless keratinocyte cells with large and flat, irregular shape and without or with a small amount of white blood cells and nuclear epithelia. The number of cornification epithelia decreased and number of nuclear epithelial cells increase in the late estrus. Diestrus is almost full of white blood cells with more mucus.

### Serum hormone analysis

After modeling or treatment with DK for one month, mouse blood was collected by orbital blood collection. The serum was obtained by centrifugation at 500 g for 5 min and then at 700 g for 5 min in a 4 ℃ centrifuge. The serum in the supernatant were stored in -80 ℃ refrigerator before analysis. We could collect about 200 µl serum every mouse. Serum levels of LH, FSH, testosterone and estradiol were detected by ELISA kit (Nanjing Jiancheng Bioengineering Institute).

### Tissue morphological and immunohistochemical analysis

The ovaries and brown adipose tissue were fixed in 4% paraformaldehyde (PFA), and dehydrated in increasing concentrations of ethyl alcohol, followed by clearing of alcohol by xylene. then the tissues were placed in a wax cylinder for overnight immersion. The embedded tissue is cut into 5-µm slices for experiments. Ovarian and brown adipose tissue sections were stained with HE staining kit (Solarbio, G1120). The morphology of ovary and brown adipose tissue was observed under a microscope. The cystic follicles and corpus luteum in the ovaries of the experimental group and the control group were counted under a microscope.

Immunohistochemistry was performed with 5 μm thickness tissue. The antibody against UCP1 (Abcam, AB10983) was incubated overnight at 4 degrees at the dilution ratio of 1:200. The antibody was incubated with anti-rabbit secondary antibody at room temperature for 1 h in the next day before developing the signal under microscope.

### Glucose tolerance test

The mice were fasted at 5 p.m. and the experiment began at 9 a.m. in the next day after 16 h, which the water intake was normal during the time. Before glucose injection, blood glucose level was measured. Glucose (Sigma, SLBP7056V) was prepared into 300 mg/ml with saline. The glucose was injected according to 5 µl /g. Blood glucose was measured at indicated timepoints. After the experiment, food was provided again.

### Insulin resistance test

The mice were fasted for 4 h (9:00–13:00) and they drank water normally. The injection volume of insulin (Macklin, I828365) was calculated according to body weight and the dosage was 1U /kg. The insulin was prepared in saline at 1U /ml. Blood glucose was firstly measured before insulin injection, after that it was measured at indicated timepoints.

### Fertility assessment

The female mice were firstly placed in the cage with a fertile male mouse (1: 2) overnight for ovulation test. Vaginal plugs were checked next morning at 9:00 to determine the ovulation and mating. The ampulla of the oviduct was punctured with a 1ml needle to observe whether there was egg mass outflow. The granulosa cells around the egg were removed by hyaluronidase for taking photos. The day when the vaginal plug was observed was defined as D1. On D8, the mice were sacrificed by cervical dislocation method, and the uterus of the mouse was removed to detect the implantation sites.

### Quantitative real-time PCR analysis

The brown adipose tissue was cut into 10 mm pieces and frozen in liquid nitrogen. Trizol (Vazyme, B2252CMA) was used to extract total RNA from the tissue according to the instructions. According to the instruction of the Reverse transcription Kit (TAKARA), 1 ng of RNA was used as the template for reverse transcription reaction. Real-time quantitative polymerase chain reaction (PCR) was performed on an ABI Q5 Real Time PCR instrument using SYBR Green (Takara) with a final volume of 10 µl. The cycle conditions were 95 ° C for 30s, 95 ° C for 5 S, and 60 ° C for 34s, 40 cycles. QPCR data was normalized using 2-ΔΔct. All expression values were normalized with GAPDH. All PCR primer sequences are listed in Supplementary Table 1.

### Bodipy staining for lipid droplet

After a month treatment with DK, frozen section of brown adipose tissue is fixed with 4% paraformaldehyde (PFA) for 15 min. After that, the slides were washed three times by PBS, then stained with Bodipy (Boer, 878557-19-8) (1:1500) for 15 min and the positive signal was observed under a fluorescence microscope.

### Triglyceride detection

50 mg of brown adipose tissue was weighed and tested according to the operation steps of Solarbio’s triglyceride detection kit (BC0625). The amount of triglyceride contained in brown adipose tissue per unit mass was obtained according to the calculation formula in the kit.

### RNA-Seq and bioinformatic analysis

The brown adipose tissue after a month treatment with DK was cut into 10 mm pieces and frozen in liquid nitrogen. Trizol (Vazyme, B2252CMA) was used to extract total RNA from the tissue according to the instructions. Purified RNA was prepared and subjected to RNA sequencing using BGISEQ-500 platform (China, BGI). RNA-seq raw data were initially filtered to obtain clean data after quality control by Trimgalore. High-quality clean data were aligned to the mouse reference genome (mm10) using STAR. Differential expression genes were normalized to fragments per kilobase of exon model per million mapped reads (RPKM) using the EdgeR 3.9 package in R with the criteria of fold change significantly greater than 2 or less than 0.5 and P < 0.05. The visualization of RNA-Seq data were done by ggplot2 package in R.

### Data analysis

GraphPad Prism 6.02 was used for data analysis. T-test was used to analyze the significance of differences between two groups and one-way analysis of variance (ANOVA) was used to analyze the statistical significance of differences between three groups or more. P value less than 0.05 indicates a significant difference. ImageViewerG was used to calculate and analyze the number of polycystic ovaries and corpus luteum in the control and experimental groups.

## Results

### Both hormone levels and fertility were severely disturbed in DHEA-induced PCOS mice

**Fig. 1:**
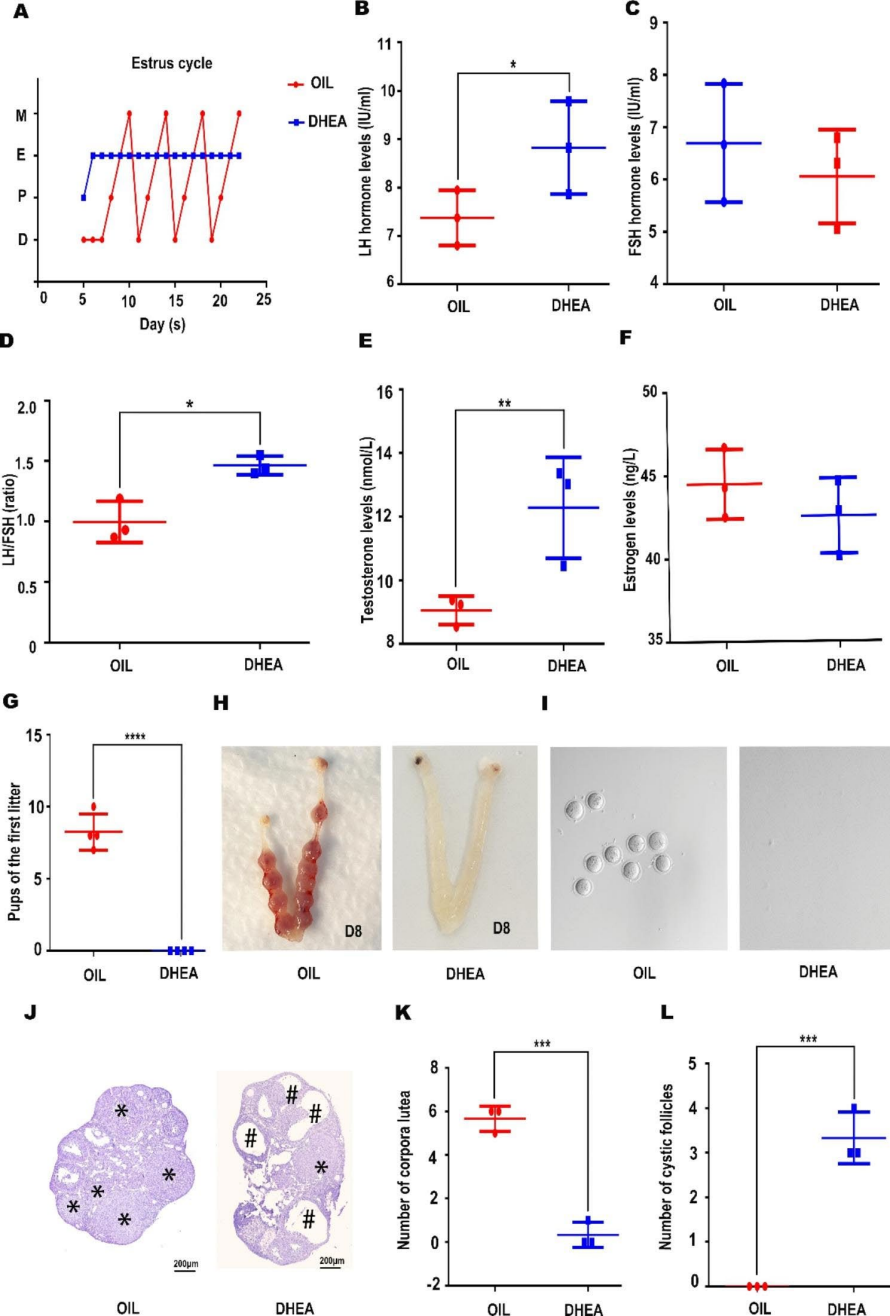
Both hormone levels and fertility are affected in DHEA-induced PCOS mice. The mice were divided into two groups (OIL, DHEA). (**A**) The analysis of estrous cycles (n = 8 per group). Mice injected with DHEA remained in estrous from the fifth day of injection. D, diestrus; E, estrus; M, metestrus; P, proestrus. (**B**, **D**, **E**) mice injected with DHEA significantly increased the concentrations of luteinizing hormone (LH) levels, as well as the LH/FSH ratio and testosterone levels, respectively, but not follicle stimulating hormone (FSH) and estrogen (**C**, **F**). Two-tailed, (n = 3 mice per group). (**G**, **H**, **I**) The fertility of mice injected with DHEA was impaired. Mice injected with DHEA have ovulation disorders. Two-tailed, (n = 3 mice per group). (**J**) The ovaries of mice injected with DHEA developed cystic follicles with corpus luteum depletion. # Indicates cystic follicles. * Indicates Corpus luteum. (**K**, **L**) The number of corpuses luteum and cystic follicles was counted, respectively. Two-tailed, *P < 0.05, **P < 0.01, ***P < 0.001,**** P < 0.0001. (n = 3 per group)

In order to investigate the therapeutic effects of DK on PCOS, we first constructed a faithful mouse model of PCOS. The PCOS model was induced by subcutaneous injection of dehydroepiandrosterone (DHEA) as reported in previous studies [23]. The mice were divided into two groups, one oil-injection group (OIL) as a control group and the other DHEA-injection group (DHEA) as an experimental group. Disturbance of the estrous cycle is a major feature of PCOS mice. To ensure the model is constructed successfully, the estrus cycle was examined. It was discovered that the experimental group was in constant estrus from the fifth day of treatment compared to control group, which showed a normal estrus cycle (Fig. 1A; Table 1). Further, serum levels of luteinizing hormone (LH) and follicle stimulating hormone (FSH) were measured, and their ratio was also analyzed. Both LH level and LH/FSH ratio were memorably higher in the DHEA group compared to control (Fig. 1B, C, D), consistent with the observation in PCOS patients [24]. We also analyzed serum testosterone and estrogen levels between the two groups. Testosterone levels were increased in the DHEA group compared to the OIL group, and estrogen displayed no significant change (Fig. 1E, F).

**Table 1:** The estrous cycle of mice with different treatment

Group	Total no.	Normal estrous cycle	Abnormal estrous cycle
OIL	8	8	0
DHEA	8	0	8

As PCOS can severely affect fertility, we next examined the mouse fertility. The fertility was remarkably impaired in the DHEA group since there were no successful pregnancies (Fig. 1G, H). We then further analyzed the phenotype and found that mice in the DHEA group failed to ovulate successfully (Fig. 1I). HE staining of the ovaries revealed the presence of multiple cystic follicles in the ovaries of the DHEA group (Fig. 1J). The number of corpora luteum in the ovaries of the DHEA group was lower and the number of cystic follicles was markedly higher compared to control group (Fig. 1K, L). Based on these data, DHEA treated mice faithfully modeled the typically impaired fertility of PCOS.

### DHEA-induced PCOS mice displayed abnormalities in glucose metabolism

**Fig. 2:**
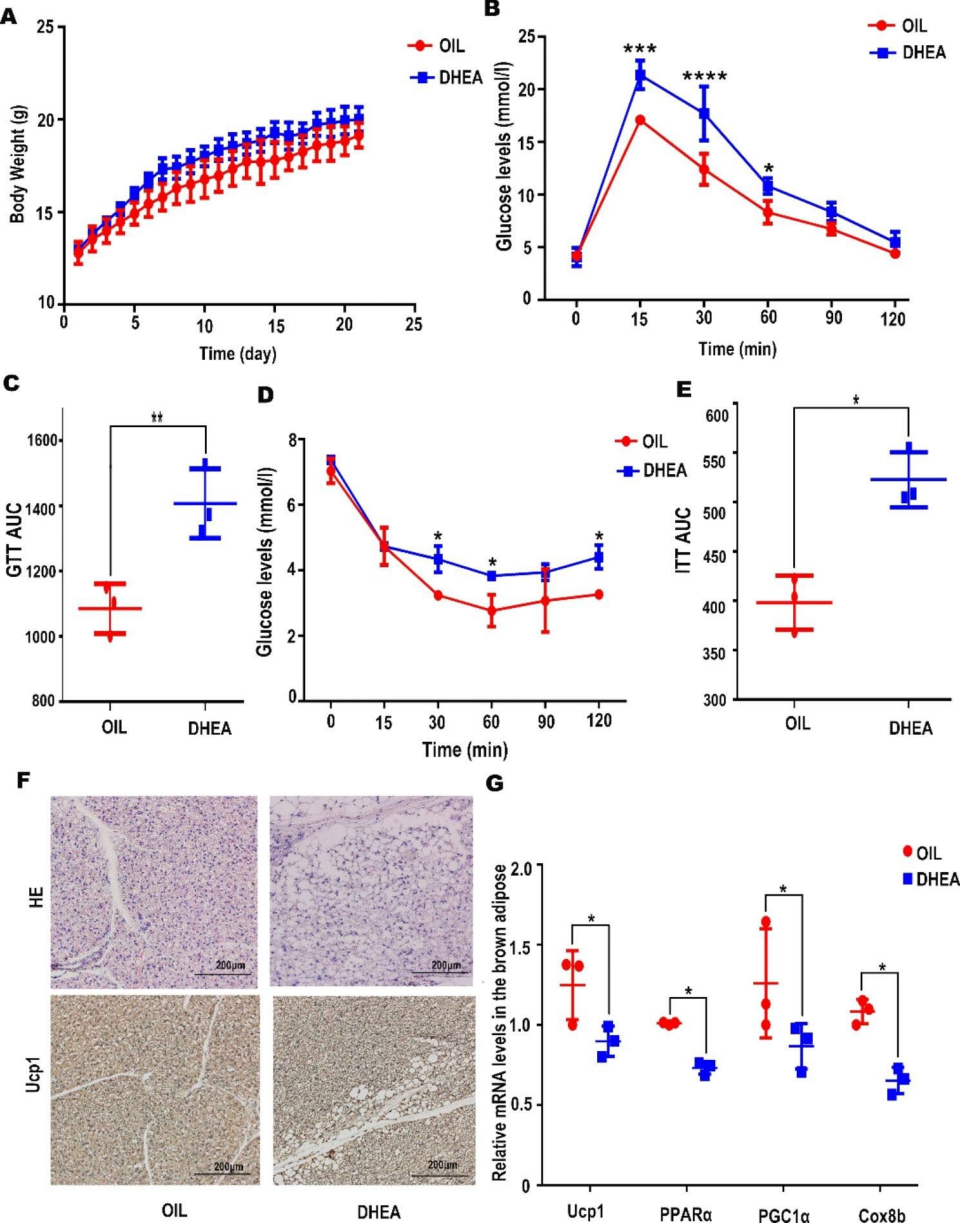
DHEA-induced PCOS mouse displayed abnormalities in metabolism. The mice were divided into two groups (OIL, DHEA). (**A**) The body weight of OIL, OIL + DK, DHEA, DHEA + DK treated mice. Number within the bar indicates the weight of mice. Two-tailed, P < 0.05,(n = 3 mice per group). (**B**,**C**) GTT assay. AUC indicates area under the curve of GTT (n = 3 mice per group). (**D**,**E**) ITT assay. AUC indicates area under the curve of ITT (n = 3 mice per group). B,C,D,E used two-tailed, P < 0.05.(**F**) Compared the OIL and DHEA groups, hismorphology and expression of protein Ucp1 in brown adipose. (**G**) Ucp1,PPARα,PGC1α,Cited1,Cox8b mRNA expression levels in the brown adipose were measured by qPCR (n = 3 per group), Two-tailed, *P < 0.05, **P < 0.01, ***P < 0.001,**** P < 0.0001

After 21 days of DHEA treatment, we first analyzed the dynamic changes of body weight between the control and experimental groups. It suggested that the body weight of mice in DHEA group was markedly higher than the control group (Fig. 2A). Another major feature of PCOS is the metabolic abnormality manifested as insulin resistance and the inability to use glucose properly [25]. Glucose tolerance (GTT) and insulin resistance (ITT) assayed was conducted in control and DHEA groups. Mice in DHEA group displayed abnormal glucose tolerance, which showed glucose intolerance and insulin resistance compared to control mice (Fig. 2B, C, D and E).

In order to investigate whether the metabolic abnormalities in the DHEA group were associated with changes in brown adipose tissue, we analyzed the morphology and functional molecule expression of brown adipose tissue. Compared to the control group, the HE staining suggested that whitening of brown adipose tissue was increased in the DHEA group since the adipose cells were more hypertrophic (Fig. 2F). UCP1 is a thermogenesis marker of brown adipose tissue [26]. Peroxisome proliferator activated receptor gamma coactivator 1 alpha (PGC1α) induces the expression of UCP1. Peroxisome proliferator activated receptor alpha (PPARα) is involved in the regulation of lipid metabolism [27], and Cytochrome c oxidase subunit 8B (Cox8b) is an enzyme that drives oxidative phosphorylation [28]. All of these molecules were consistently down-regulated in the DHEA group (Fig. 2G). The above data supported the metabolic disturbance in the DHEA-induced PCOS mice and defective brown adipose tissue activation with lower capacity of thermogenesis.

### DK prominently improved impaired fertility in the PCOS mice

**Fig. 3:**
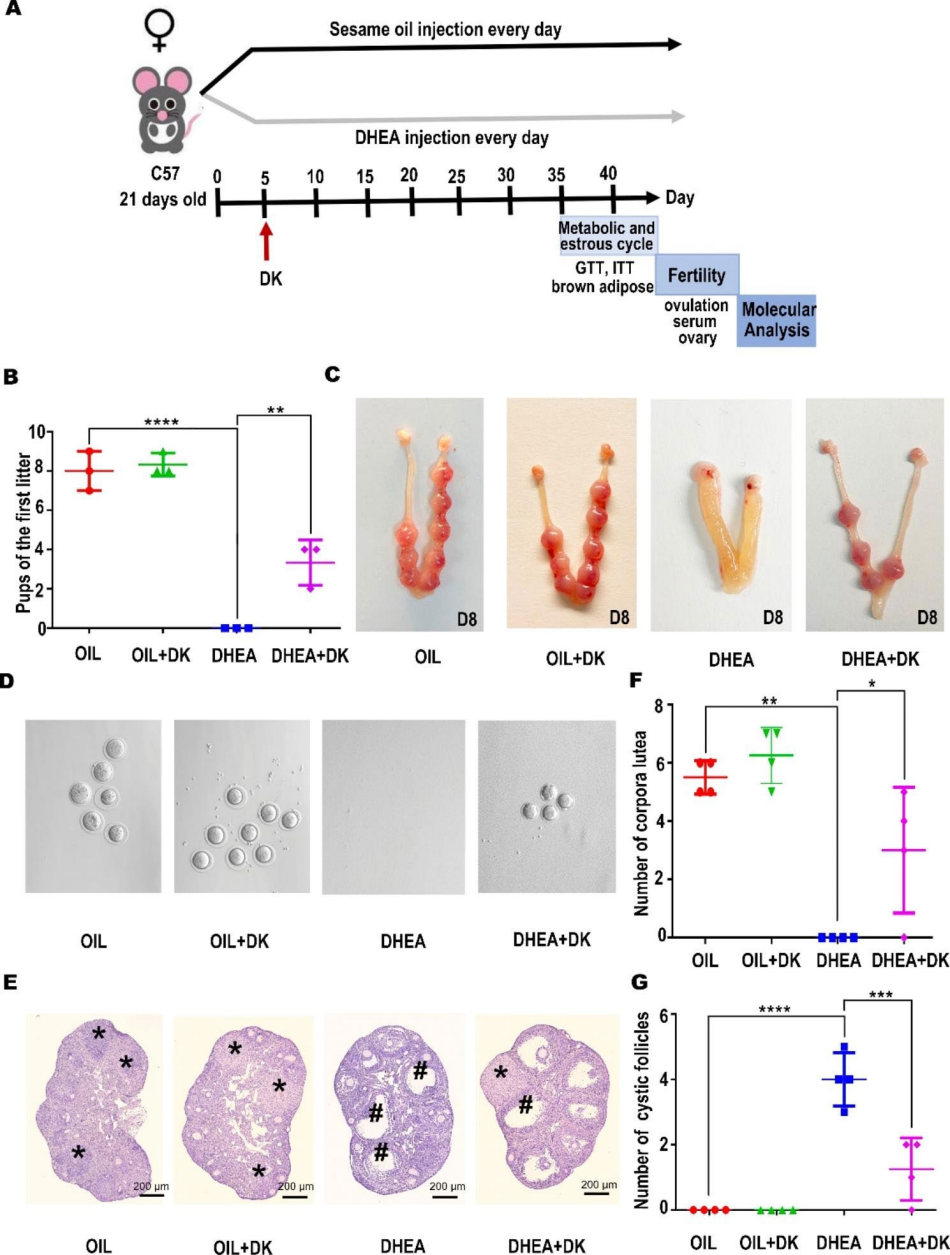
DK prominently improves impaired fertility in the PCOS mice. The mice were divided into four groups (OIL, OIL + DK, DHEA, DHEA + DK). (**A**) Schematic diagram of early treatment strategies. (**B**, **C**) DK can effectively improve the reproductive abnormalities of PCOS mice. (**D**) The mice were tested for ovulation on the day of vaginal plug. (**E**) DK significantly increased the number of corpuses luteum and decreased the number of cystic follicles in PCOS mice. # Indicates cystic follicles. * Indicates Corpus luteum. (**F**, **G**) The statistics of the corpus luteum and the follicles, respectively. All data were analyzed by one-way ANOVA with Tukey’s post hoc test. *P < 0.05, **P < 0.01, ***P < 0.001,**** P < 0.0001. n = 3 per group

Next, we used established DHEA mouse model to investigate the therapeutic effect of DK on PCOS by measuring its relevant metabolic and reproductive parameters. The experimental strategy of DK in the treatment of PCOS mice was shown in Fig. 3A. On the fifth day of DHEA treatment, the estrous cycle of the mice had changed. Subsequently, DK treatment started from the fifth day of DHEA injection till to the sacrifice. The mice were divided into four groups (OIL, OIL + DK, DHEA and DHEA + DK) to assess the alleviation of infertility in PCOS mice after DK treatment. Impressively, PCOS mice with DK treatment could give birth normally, despite having fewer offsprings (Fig. 3B, C). As the main reproductive condition of PCOS is ovulation disorder, the ovulation of the mice was examined. The PCOS mice with DK treatment recovered the ovulation ability, though the number of oocytes expelled was lower than in OIL group (Fig. 3D). Consistently, the morphology of the ovaries showing cystic follicles in the DHEA + DK group was restored after DK treatment (Fig. 3E). The number of corpora luteum (Fig. 3F) in the ovaries of PCOS mice was significantly increased after DK treatment and the number of cystic follicles was remarkably reduced (Fig. 3G). Based on these data, the DK could effectively improve the symptom of impaired fecundity, partially restore ovulation and normal calving in PCOS mice. It could also availably ameliorate the pathological characteristics of ovarian cystic follicles.

### DK restored the abnormal hormone levels and estrus cycle in the PCOS mice

**Fig. 4:**
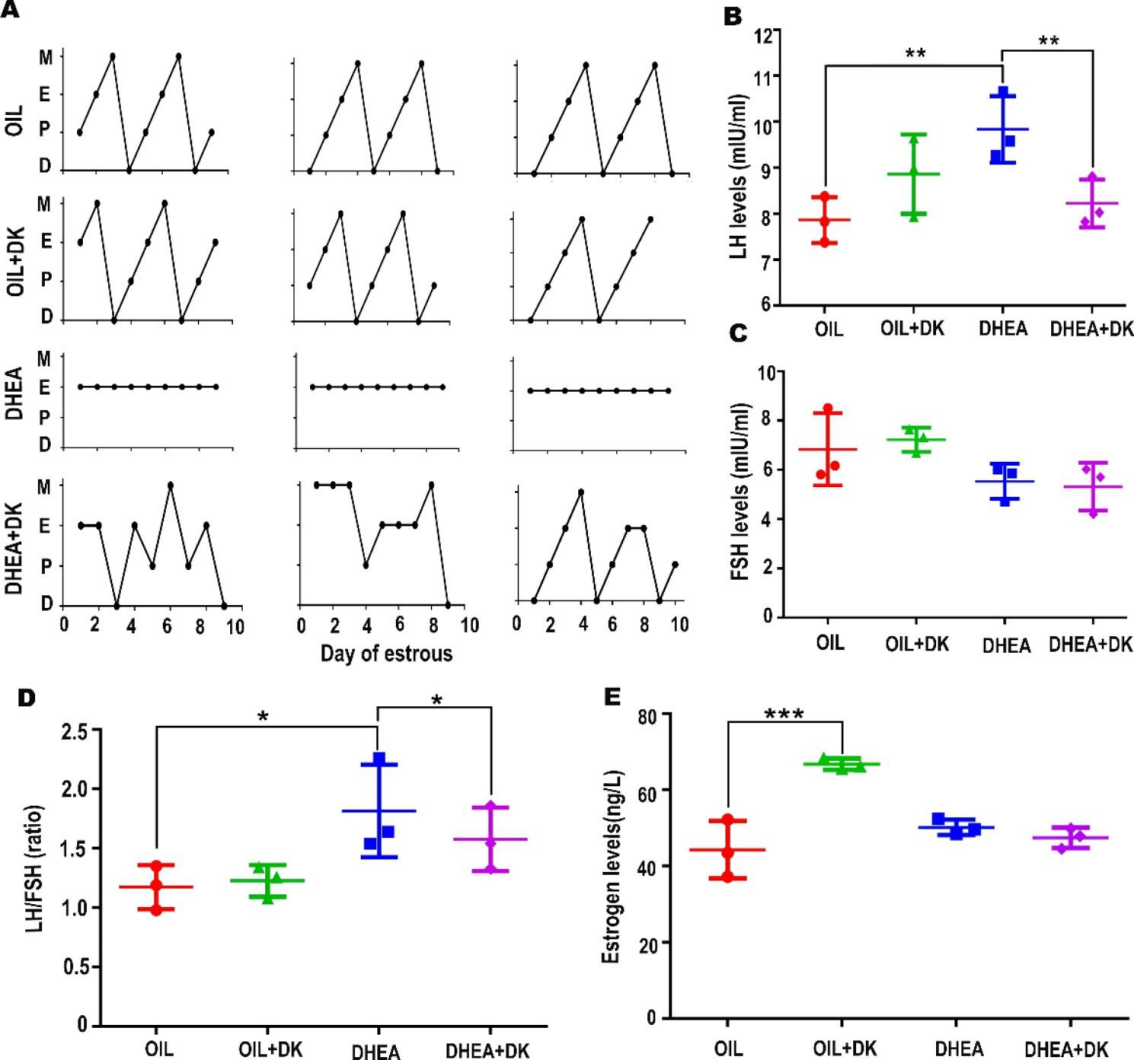
DK restores the abnormal hormone levels and estrus cycle in the PCOS mice. The mice were divided into four groups (OIL, OIL + DK, DHEA, DHEA + DK). (**A**) DK could partly reverse abnormal estrous cycles in the PCOS mice compared with abnormal estrous cycles in the DHEA treated mice. **D**, diestrus; **E**, estrus; **M**, metestrus; **P**, proestrus. (**B**, **D**) DK can significantly reduce the LH level and LH/FSH ratio caused by PCOS. (**C**, **E**) The serum level of follicle stimulating hormone (FSH) and estrogen, respectively. All data were analyzed by one-way ANOVA with Tukey’s post hoc test. *P < 0.05, **P < 0.01,***P < 0.001 n = 3 per group

The regular estrus cycle determines the faithful fertility in female, which is disturbed in PCOS mice. Based on the fertility restored in DK treated mice, we intended to determine whether the estrous cycle was rescued. Compared to the DHEA mice, the estrous cycle of the DHEA + DK mice did not remain consistent in estrus and began to recover (Fig. 4A; Table 2). Subsequently, we examined the expression of relevant hormone levels. We found an increase in LH as well as in the LH/FSH ratio, indicating that there was also some recovery in their hormone status after DK treatment (Fig. 4B, C, D). Estrogen levels did not differ between the four groups (Fig. 4E). The above data revealed that besides the recovery of fertility abnormalities in DK-treated PCOS mice, their estrous cycle and related hormone levels were also restored to a certain extent.

**Table 2:** The effect of Dingkun Pill on estrous cycle

Group	Total no.	Normal estrous cycle	Abnormal estrous cycle
OIL	6	6	0
OIL + DK	6	6	0
DHEA	6	0	6
DHEA + DK	6	4	2

### DK significantly ameliorated metabolic abnormalities in the PCOS mice

**Fig. 5:**
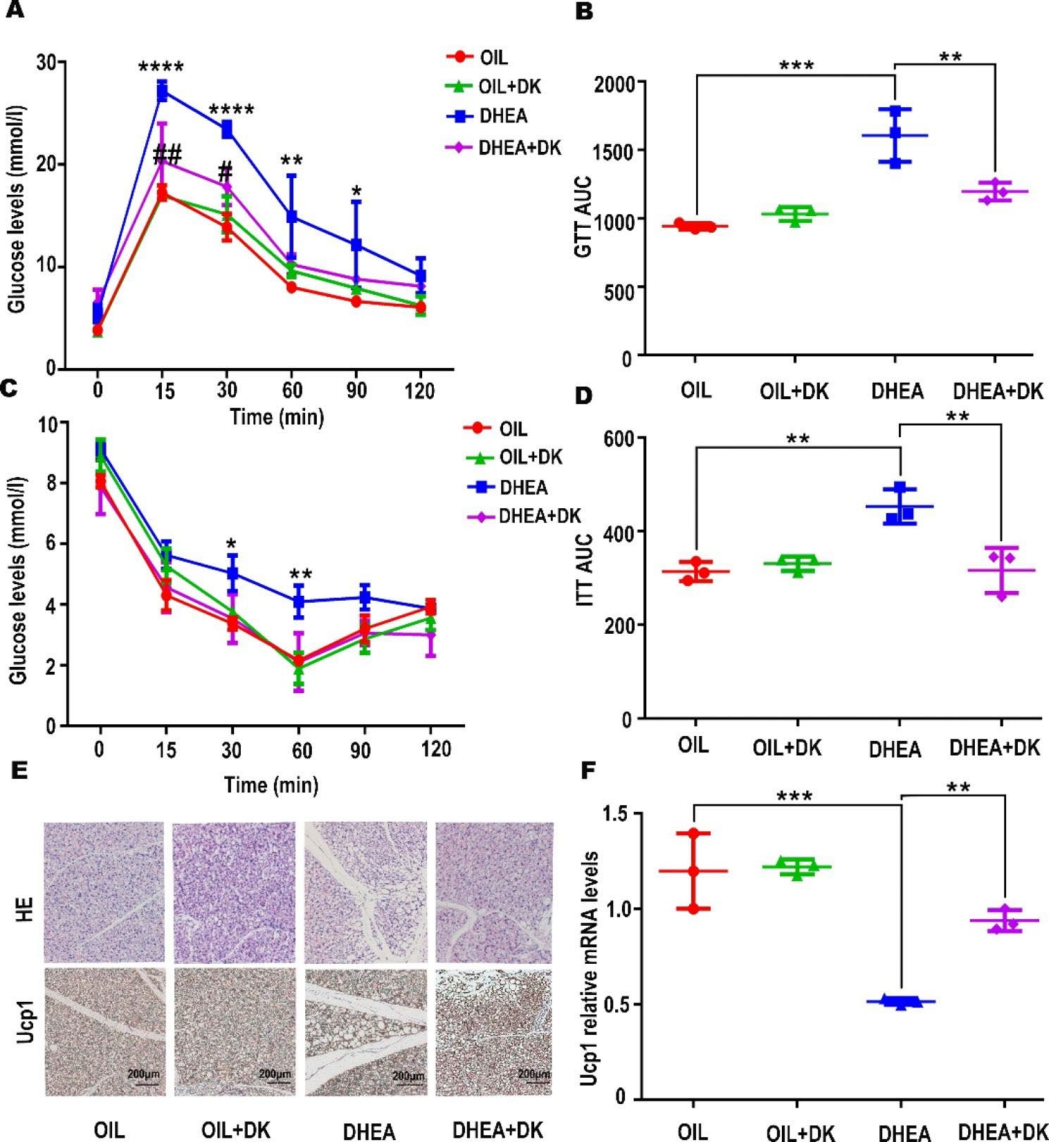
DK significantly improves the characterization of metabolic abnormalities in the PCOS mice. The mice were divided into four groups (OIL, OIL + DK, DHEA, DHEA + DK). (**A**, **B**, **C**, **D**) DK has a certain therapeutic effect on the abnormal glucose tolerance and insulin resistance caused by DHEA injection, respectively. * means the significant differences of glucose level of DHEA compared to OIL group in the A and C. # means the significant differences of glucose level of DHEA + DK compared to OIL group in the A. B and D indicate area under the curve of GTT and ITT, respectively. Data were analyzed by one-way ANOVA with Tukey’s post hoc test. P < 0.05 n = 4 per group. (**E**) DK significantly reversed the whitening of brown adipose and the decrease of Ucp1 protein. (**F**) Ucp1 mRNA expression levels in the brown adipose were measured by qPCR (n = 3  per group). Data were analyzed by one-way ANOVA with Tukey’s post hoc test. *P < 0.05, **P < 0.01, ***P < 0.001, **** P < 0.0001, #P < 0.05, ##P < 0.01

Although DK did not restore the weight gain caused by DHEA injection (Fig S1A), we found that the DHEA + DK group showed recovery of insulin resistance and some improvement in abnormal glucose tolerance compared to DHEA group, and DK itself did not impact the metabolic homeostasis in OIL and OIL + DK group (Fig. 5A, B, C, D). To test whether the activation status of brown adipose tissue was changed in related with the systematic metabolism changes, we examined the degree of whitening in the brown adipose tissue and the expression of Ucp1. We found a reduction in the amount of whitened brown adipose tissue after DK treatment, suggesting a partial improvement of brown adipose tissue in PCOS mice after DK treatment (Fig. 5E). Consistently, the expression of Ucp1 was also significantly increased in the DHEA + DK group (Fig. 5F). This suggested that the activity of brown adipose tissue was improved to some extent in the DHEA-induced PCOS mice after the DK treatment. We found that DK treatment was effective in ameliorating the metabolic abnormalities in DHEA induced PCOS mice. Thus, recovery of brown adipose tissue metabolism after DK treatment at least contributed the improvement of global metabolic abnormalities in PCOS mice.

### DK treatment in PCOS mice activated the brown adipose metabolism and thermogenesis

As a metabolic and thermogenic organ, brown adipose plays a pivotal role in the regulation of metabolic homeostasis throughout the body. Therefore, we further explore whether the therapeutic effect of DK on PCOS is related to the recovery of brown adipose metabolism and thermogenesis. RNA-Seq sequencing was performed on brown adipose from three groups of mice (OIL, DHEA, DHEA + DK). From the sequencing results of brown adipose in different treatments, it showed that DHEA treated mice showed a significant decrease in brown adipose related thermogenesis markers Ucp1, PGCLα, and Cox8b compared with the OIL group.

**Fig. 6:**
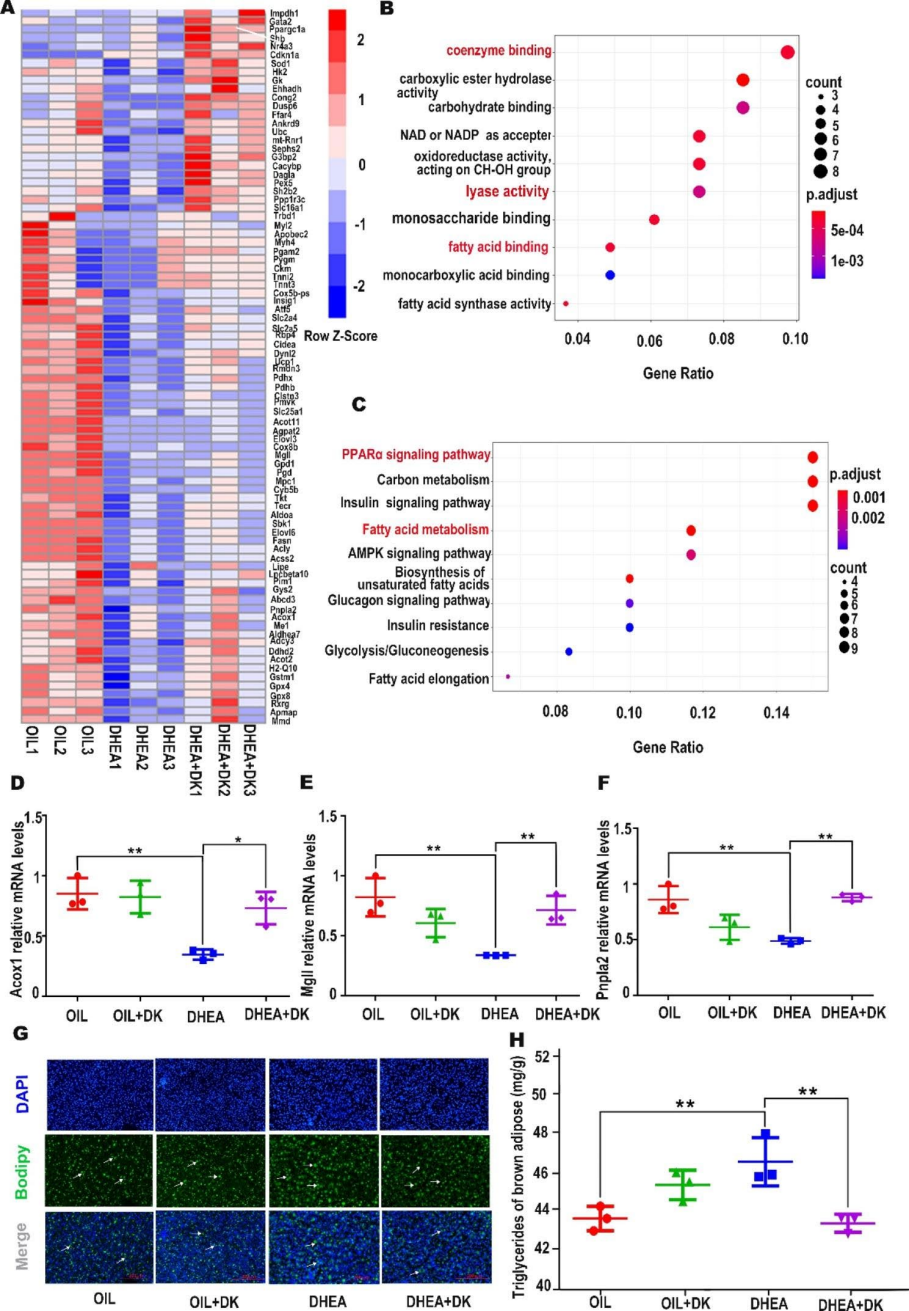
DK treatment in PCOS mice activate the brown adipose metabolism and thermogenesis. (**A**) Heat map shows the expression level of differentially upregulated genes in OIL, DHEA and DHEA + DK groups. (**B**, **C**) B and C represent GO and KEGG analysis of the differentially expressed genes in DHEA and DHEA + DK groups, respectively. P values were determined by two-tailed Wilcoxon rank-sum test. (**D**, **E**, **F**) QRT-PCR analyzes the mRNA expression of lipid metabolism related Acox1, Mgll, Pnpla2 among OIL, OIL + DK, DHEA and DHEA + DK groups. (**G**, **H**) Lipid droplet content among OIL, OIL + DK, DHEA and DHEA + DK groups. All data were analyzed by one-way ANOVA with Tukey’s post hoc test. *P < 0.05, **P < 0.01, n = 3 per group

Genes associated with metabolism include PPARα, Pnpla2 and Mgll, which encode the lipase that sequentially lysis the triglyceride [29], were significantly downregulated in the DHEA group compared with OIL group (Fig. 6A). Impressively, this expression change tendency was recovered after treatment with DK. Similar expression changes exist for Peroxisomal acyl-coenzyme A oxidase 1 (Acox1), which is involved in the initiation and rate-limiting steps of peroxisome-oxidation of linear and unsaturated long-chain fatty acids [30].

To reveal the global molecular changes of DK treatment, we further performed Gene Ontology and Kyoto Encyclopedia of Genes and Genomes analysis to explore the most significantly influenced biological event after DK treatment. We found that biological processes and signaling pathways related to lipid metabolism were enriched (Fig. 6B, C). We further verified the expression of key enzymes in lipid metabolism by real-time quantitative PCR, such as Pnpla2, Mgll, Acox1, PPARα, PGCLα, and Cox8b. The expression of these molecules decreased after DHEA treatment, which would disturb the lipid homeostasis and lead to brown adipose tissue whitening, and there was a certain degree of recovery after DK treatment (Fig. 6D- F, Fig S2 A-C). Since lipid droplets mainly exist in cells in the form of triglyceride, we then detected the level of triglyceride in brown adipose tissue. Compared with the control group, the DHEA group accumulated more lipid droplets in brown adipose with a larger shape (Fig. 6G, H), consistent with the lower mobilization of triglyceride due to the downregulation of lipase. Interestingly, the accumulation of lipid droplets in the brown adipose of PCOS mice was restored after DK treatment. In summary, the therapeutic effect of DK on PCOS was at least partially attributed to the recovery of brown adipose metabolism and thermogenesis.

## Discussion

**Fig. 7:**
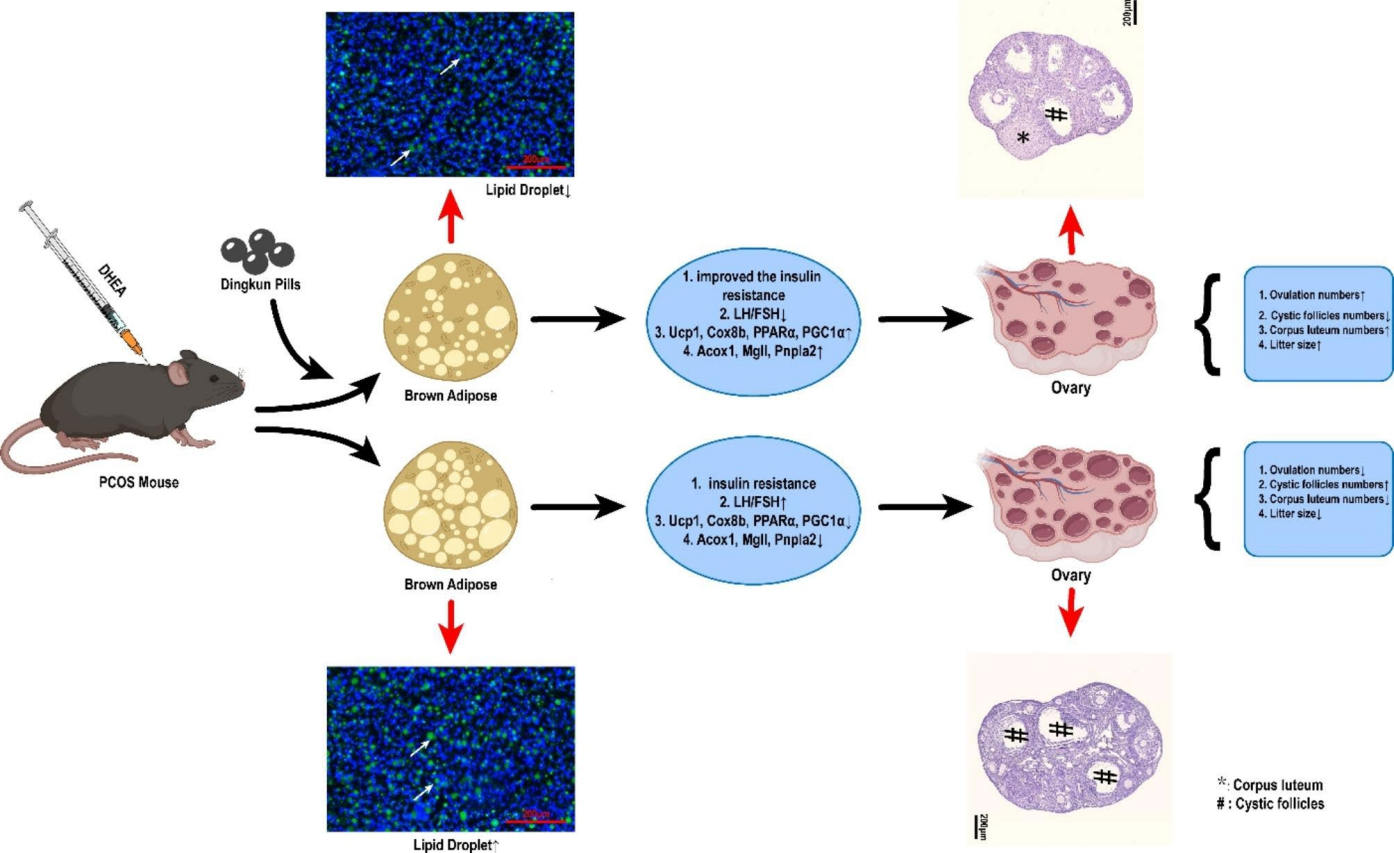
Schematic of DK therapy PCOS mice by activating the brown adipose metabolism and thermogenesis

In this study, we constructed a DHEA induced mouse PCOS model, and found that DK has a therapeutic effect on PCOS. DK can significantly improve the damaged fecundity of PCOS mice, and restore both the abnormal estrous cycle and reproductive hormone level to a great extent in PCOS mice. Impressively, DK can also alleviate metabolic abnormalities caused by PCOS. As an important metabolic organ, brown adipose plays an important role in regulating the whole-body metabolism. Indeed, we found that DHEA induced abnormal lipid accumulation in brown adipose, similar to the report in white adipose tissue [31]. Moreover, the thermogenesis and metabolic functions of brown adipose in PCOS mice were improved after DK treatment. This suggests that the therapeutic effects of DK on PCOS may at least partially due to brown adipose activation. DK may play a certain therapeutic role in PCOS by acting on brown adipose to improve its abnormal lipid metabolism and thermogenesis (Fig. 7). Whether the components in DK or in vivo metabolites directly target the brown adipose tissue need further exploration based on the identification of the effective molecular in vivo after DK treatment, and this will also support the directly functional and mechanism study in the brown adipocyte. A recent report has demonstrated that Ginseng extract can regulate the serum myristoleic acid level to regulate the brown adipose activation through the gut microbiota [32].

Besides reproductive manifestations of anovulation, cystic follicles and infertility, as a common endocrine disorder, PCOS has many endocrine metabolic phenotype such as insulin resistance and dyslipidemia [33]. Long-term lipid metabolism disorders can seriously endanger women’s health. Most patients with PCOS develop obesity, which is mainly due to lipids accumulation and reduction of lipid activation and oxidation [34]. The most efficient way to store lipids is to de novo synthesize triglycerides (TG) in adipose tissue [30]. Abnormalities in lipid metabolism of PCOS patients are mainly manifested by an increase in TGs [35]. The RNA-Seq sequencing results of brown adipose tissue showed that the expression of lipolysis related genes was down-regulated, which was somewhat alleviated after DK treatment (Fig. 6A-C). Changes in lipolysis related genes affect the metabolism of TGs. TGs are firstly lipolyzed and then activated to form acyl-CoA before entering the mitochondria for complete oxidation [36]. A variety of lipolysis-related enzymes are involved in this process. Adipose TG lipase (ATGL) is a rate-limiting enzyme involved in the initiation of cytoplasmic lipid droplets (LDs) TG hydrolysis into diacylglycerol and fatty acids in cytoplasmic lipid droplets (LDs), which efficiently and specifically clears away intracellular TGs [37, 38]. MGs produce glycerol and fatty acids under the catalyzation of MG lipase (MGL) [39]. Through our data, it shown that there was obvious downregulation of both ATGL and MGL in brown adipose tissue of PCOS mice (Fig. 6D-F). The decreased expression of lipolytic enzyme in PCOS mice indicates abnormal triglyceride metabolism. Exactly, our results suggest that PCOS mice do have abnormal accumulation of lipids in brown adipose tissue. By fluorescence staining of lipid droplets, it was found that the brown adipose tissue of PCOS mice accumulated more lipid droplets with large size, which may be the cause of abnormal metabolism. The expression of ATGL and MGL was restored after DK treatment. It is suggested here that the therapeutic effect of DK on PCOS mice may be achieved by regulating the expression of lipolytic-related enzymes in brown adipose. Further research is needed to explore how these lipases are regulated by the DK in brown adipose tissue.

The fatty acids produced by the lipolysis of triglycerides can be activated intracellular to form acyl-CoA, and then transported to mitochondria for β-oxidation and finally to form acetyl-CoA, FADH2 and NADH. Acetyl-CoA enters the tricarboxylic acid cycle for further metabolism to supply energy for the body. FADH2 and NADH undergo oxidative phosphorylation to produce ATP [40]. Peroxisome proliferator activated receptor alpha (PPARα) is involved in the regulation of lipid metabolism [27]. PPARα induces expression of mitochondrial acyl-CoA dehydrogenases, leading to an increase in fatty acid oxidation and acetyl-CoA production [41]. We found that PPARα expression was decreased in brown adipose tissue of PCOS mice (Fig S2B), indicating abnormal lipid metabolism. After DK treatment, the abnormal lipid metabolism was significantly improved accompanied with the restored expression of PPARα, suggesting that DK may regulate the expression of PPARα to improve the abnormal lipid metabolism. Moreover, brown adipose tissue is also a thermogenic organ and the chemical energy produced through the oxidative phosphorylation cannot synthesized the ATP, but produced heat through the respiratory chain in the mitochondria. Uncoupling protein 1 (UCP1) is a heat producing marker of brown adipose tissue. UCP1 is involved in oxidative phosphorylation reactions in brown adipose tissue mitochondria. It disrupts the proton concentration gradient through uncoupling, thereby affects ATP production and allows energy to be dissipated as heat [42]. Peroxisome proliferator activated receptor gamma coactivator 1 alpha (PGC1α) induces the expression of UCP1. Cytochrome c oxidase subunit 8B (Cox8b) is an enzyme that drives oxidative phosphorylation [28]. All of these molecules were consistently down-regulated in the PCOS mice. These results indicate that the thermogenesis of brown adipose tissue in PCOS mice is impaired due to both the decreased oxidative phosphorylation and downregulation of UCP1. However, the expression of thermogenic molecules in brown adipose tissue of PCOS mice was up-regulated after DK treatment, indicating that the thermogenic function of PCOS mice had been restored to a certain extent.

Excessive lipid accumulation could aggravate inflammation and insulin resistance, which affects the function of the ovary to promote the production of androgens [43, 44]. There is a close relationship between lipid metabolism and ovarian function, and research in this area has received increasing attention in recent years [45]. Excessive accumulation of lipids leads to the production of cholesterol, which is a precursor to steroid hormone production and thus promotes androgen hyperplasia. Excess androgen inhibits follicle development and maturation [46]. The increase of Anti-muller hormone (AMH) in PCOS patients will inhibit the expression of aromatase, which can catalyze the generation of androgens into estrogen [47]. However, the loss of estrogen and excessive androgens in the body will lead to the decrease of the expression of enzymes related to lipolysis, which promote the accumulation of lipid, forming a positive feedback loop to deteriorate the reproductive and metabolism disorder [48]. Daine-35 is a kind of estrogen drug for the treatment of PCOS by antagonizing androgens [49]. Combined use of Diane-35 and metformin improves PCOS in rat model possibly via regulating metabolism [50]. Our previous report has demonstrated that DK can increase the effects of estrogen signal in the endometrium [51], whether the similar mechanism is functional for alleviating metabolic abnormalities need the further exploration.

In addition to its metabolic function, brown adipose tissue can also act as an endocrine organ, secreting proteins to regulate the body’s homeostasis. Adiponectin is a protein secreted by brown adipose tissue. It has been reported that it is significantly decreased in the serum of PCOS patients. After transplantation of brown adipose tissue, the serum adiponectin level of PCOS can be increased to achieve therapeutic effects [14]. As a potential endocrine intermediate, C-X-C motif chemokine ligand-14 protein (CXCL14) was down-regulated in PCOS. The activation of brown adipose tissue by ginsenoside compound K treatment, could restored the CCXCL14 to normal level and ameliorate PCOS [52]. However, in our study, there was no significant change of both adiponectin and CXCL14 in mRNA level after DK treatment, so there may be other secretory factors to mediate the DK’s beneficial effect on other tissues, which need further research.

In summary, through our research, we revealed that DK can efficaciously restore the abnormal metabolism in PCOS and improve the disordered estrous cycle of PCOS mice to a certain extent. It also can successfully reduce the number of cystic follicles in the ovaries and promote the successful ovulation of the ovaries in PCOS mice. Moreover, brown adipose is likely to be a target organ for DK in the treatment of PCOS, but its potential mechanism needs further research and more experimental data to support. The boundedness of this study is that it mainly focuses on mice, so more clinical data are needed to estimate the efficacy and dose of DK in patients with PCOS.

## Conclusions

DK has therapeutic effect on PCOS in DHEA treated mice and the brown adipose tissue is at least one critical target organ to alleviate the PCOS. This is achieved by not only regulating the lipid mobilization of brown adipose, but also restoring its thermogenic function.

### Electronic supplementary material

Below is the link to the electronic supplementary material.

**Supplementary Material 1: Figure S1**: DK significantly improves the characterization of metabolic abnormalities in early PCOS mice. **Figure S2**: DK treatment recover brown adipose metabolism and thermogenesis though normalization of lipid homeostasis.

## Data Availability

All data are available via the corresponding authors.
