# Supplementary material for: Dingkun pill alleviates metabolic abnormalities in polycystic ovary syndrome through brown adipose tissue activation
Source: J Ovarian Res. 2023 Aug 26;16:176. doi: 10.1186/s13048-023-01215-0 (PMC10463533; doi:10.1186/s13048-023-01215-0)
Supplement: Supplementary file 1 — Supplementary Material 1: Figure S1: DK significantly improves the characterization of metabolic abnormalities in early PCOS mice. Figure S2: DK treatment recover brown adipose metabolism and thermogenesis though normalization of lipid homeostasis. [file 13048_2023_1215_MOESM1_ESM.docx]

**
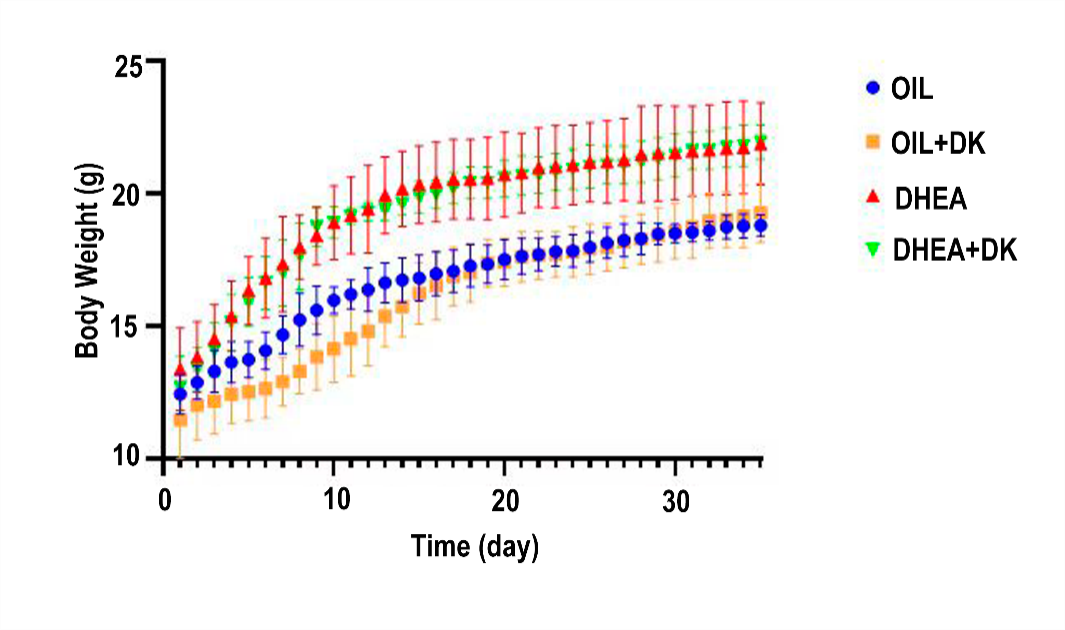
Supplementary Figures with legends：**

**Figure S1: DK significantly improves the characterization of metabolic abnormalities in early PCOS mice.** The mice were divided into four groups (OIL, OIL+DK, DHEA, DHEA+DK). The body weight of OIL, OIL+DK, DHEA, DHEA+DK. Number within the bar indicates the weight of mice. One-way ANOVA with Tukey’s post hoc test，P<0.05,n= 3 mice per group.


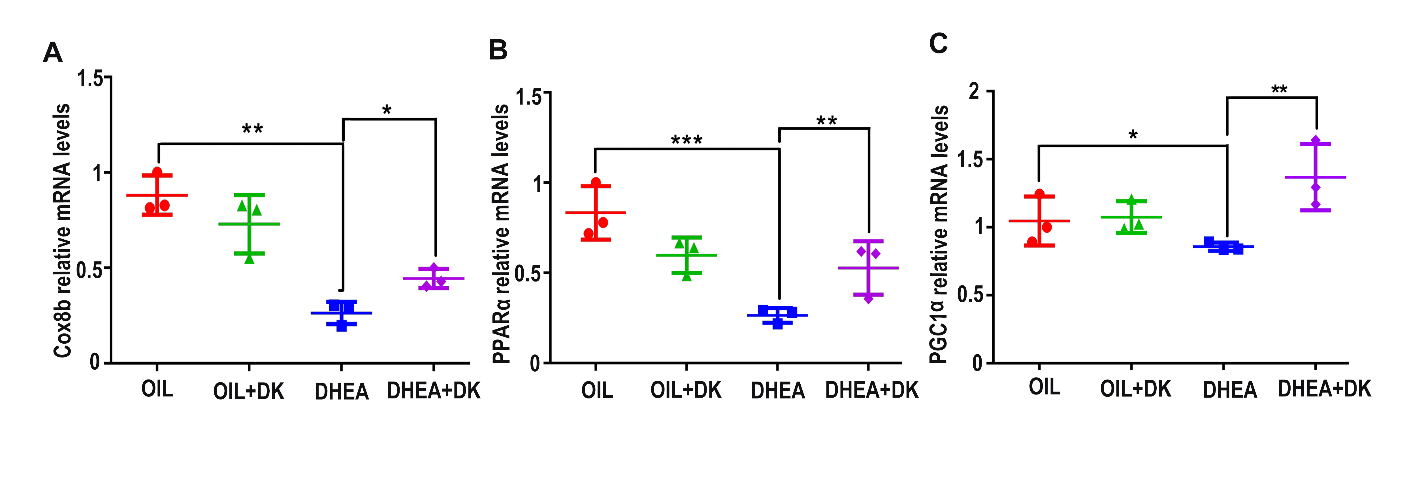


**Figure S2: DK treatment recover brown adipose metabolism and thermogenesis though normalization of lipid homeostasis.** The mRNA expression of thermogenic genes Cox8b (A), PPARα (B) and PGCL1α (C) in brown adipose respectively. All dates were analyzed by one-way ANOVA with Tukey’s post hoc test. *P<0.05, **P<0.01, ***P<0.001, n = 3 per group.
